# Diversity and community structure of aerobic anoxygenic phototrophic bacteria are shaped by the deep chlorophyll maximum

**DOI:** 10.1093/ismeco/ycag076

**Published:** 2026-03-26

**Authors:** Carlota R Gazulla, Isabel Ferrera, Vanessa Balagué, Carolina Marín-Vindas, Alba González-Vega, José Escánez-Pérez, Eugenio Fraile-Nuez, Jesús M Arrieta, Josep M Gasol, Olga Sánchez

**Affiliations:** Departament de Genètica i Microbiologia, Universitat Autònoma de Barcelona, 08193 Bellaterra, Barcelona, Spain; Departament de Biologia Marina i Oceanografia, Institut de Ciències del Mar (ICM)-CSIC, 08003 Barcelona, Spain; Centro Oceanográfico de Málaga, Instituto Español de Oceanografía (IEO)-CSIC, 29002 Málaga, Spain; Centro Oceanográfico de Málaga, Instituto Español de Oceanografía (IEO)-CSIC, 29002 Málaga, Spain; Departament de Biologia Marina i Oceanografia, Institut de Ciències del Mar (ICM)-CSIC, 08003 Barcelona, Spain; Universidad Nacional, Estación de Biología Marina, Laboratorio de Microbiología Marina (LaMMar), 60105 Punta Morales, Puntarenas, Costa Rica; Centro Oceanográfico de Canarias, Instituto Español de Oceanografía (IEO)-CSIC, 38180 Santa Cruz de Tenerife, Spain; Centro Oceanográfico de Canarias, Instituto Español de Oceanografía (IEO)-CSIC, 38180 Santa Cruz de Tenerife, Spain; Centro Oceanográfico de Canarias, Instituto Español de Oceanografía (IEO)-CSIC, 38180 Santa Cruz de Tenerife, Spain; Centro Oceanográfico de Canarias, Instituto Español de Oceanografía (IEO)-CSIC, 38180 Santa Cruz de Tenerife, Spain; Departament de Biologia Marina i Oceanografia, Institut de Ciències del Mar (ICM)-CSIC, 08003 Barcelona, Spain; Departament de Genètica i Microbiologia, Universitat Autònoma de Barcelona, 08193 Bellaterra, Barcelona, Spain

**Keywords:** aerobic anoxygenic phototrophic (AAP) bacteria, deep chlorophyll maximum (DCM), Atlantic Ocean, *pufM* gene, Luxescamonaceae

## Abstract

The surface ocean exhibits strong vertical gradients in light irradiance, nutrients, and temperature, shaping the phytoplankton distribution, which often defines a deep chlorophyll maximum (DCM). Aerobic anoxygenic phototrophic (AAP) bacteria inhabit the euphotic zone, with their abundances generally following the chlorophyll *a* variability. While AAP bacterial communities are known to differ across regions with contrasting environmental conditions, their vertical distribution remains poorly understood. We hypothesized that the diversity and community structure of AAP bacteria vary across the vertical gradient, in relation to changes in environmental variables and following the DCM profile. To test this hypothesis, we studied the composition of AAP communities at different depths along the DCM structure in the South and Central Atlantic Ocean, by means of amplicon sequencing of the *pufM* gene. The results show significant differences in richness, community structure, and taxonomic composition of samples from different layers of the DCM, highlighting the dependence of AAP bacteria on its structure. Remarkably, the use of primers with broad phylogenetic coverage enabled the recovery of several AAP phylogroups previously detected only through metagenomics. We show that they represent a significant fraction of marine AAP communities, provide clues about their ecological preferences, and confirm their association with the family *Candidatus* Luxescamonaceae.

## Introduction

The stratified pelagic sunlit ocean exhibits pronounced gradients of temperature, salinity, and light irradiance, undergoing relevant changes across tens of meters in the vertical scale. With increasing depth, there is a decline in light intensity and a rise in nutrient concentrations. In stratified oceanic waters, the zone corresponding to the upper nutricline where there is still sufficient light to support photosynthesis harbors the deep chlorophyll maximum (DCM) [1, 2]. The DCM appears as the most distinctive vertical structure that can be observed in stratified waters, situated above the pycnocline and intricately linked to the nutricline [3, 4]. This dynamic layer varies across both space and time [1] and concentrates a substantial fraction of ocean primary productivity [5–7]. Detailed characterization of the fine vertical distribution of phytoplankton groups within the DCM has uncovered its nonuniform structure, revealing the preference of specific phytoplankton groups toward distinct ecological niches [8, 9]. Considering that heterotrophic bacteria rely on phytoplankton-derived organic matter, the structure of the DCM also influences the vertical distribution of different heterotrophic groups. In fact, several studies have compared the microbiota in surface and DCM layers and observed differences in cell abundances, as well as in taxonomic and functional richness [10–12]. However, fine vertical profiling of bacterioplankton along the DCM is limited, with only a handful of studies conducted so far [13, 14, 15].

Besides phototrophs and heterotrophs, marine prokaryotic communities include photoheterotrophs, i.e. proteorhodopsin (PR)-containing bacteria and aerobic anoxygenic phototrophic (AAP) bacteria. These two groups exhibit different distributions in surface waters: PR-containing bacteria are abundant in oligotrophic systems [16–18] and AAP bacteria thrive better in more eutrophic waters [19–22]. Within the euphotic zone, bacteriochlorophyll *a* (BChl *a*)—the photosynthetic pigment of AAP bacteria—and chlorophyll *a* (Chl *a*) occupied similar niches along various trophic gradients [17]. In addition, the distribution of AAP cells has been observed to be tightly coupled with Chl *a* and the abundance of cyanobacteria and phototrophic picoeukaryotes [20]. Despite the growing information on the vertical distribution of AAP bacteria, there is little information about the diversity of their communities within the euphotic zone, as most studies have focused on their horizontal variation across the surface ocean [23–30]. These studies show that, in surface waters, AAP communities differ across areas with contrasting temperatures, salinity, and chlorophyll levels. Notably, the only study examining vertical differences in AAP communities found that populations above the DCM were significantly more similar to each other than to those within or below it [31]. In terms of diversity, AAP communities are typically composed mainly of Alpha- and Gammaproteobacteria. However, a recent analysis comparing amplicon and metagenomic approaches to study the diversity of the *pufM* gene, the genetic marker of this group, showed that a significant fraction of AAP communities consists of uncultured groups, previously missed in PCR-based studies [32]. Metagenomic analyses have revealed new AAP clades, such as *Candidatus* Luxescamonaceae, which may have the ability to fix carbon [33, 34], and are part of underrepresented uncultured groups distributed across the surface global ocean [32].

In this study, we aimed to assess whether AAP community composition varies along the vertical gradient associated with the DCM. We evaluated the composition of AAP communities by collecting samples at multiple depths across the DCM structure in areas characterized by differing productivity levels in a latitudinal transect spanning the South and Central Atlantic Ocean. The transect encompassed DCM structures of diverse nature, ranging from cold waters with shallow DCMs to warm waters where the DCMs extended below 100 m. AAP communities were investigated through amplicon sequencing of the *pufM* gene using a revised primer set [32]. These primers display a high phylogenetic coverage and minimize primer biases, allowing for a comprehensive representation of AAP communities, including uncultured groups such as *Ca*. Luxescamonaceae, thereby improving our understanding of their ecological distribution patterns.

## Material and methods

### Sample collection

The Poseidon Expedition, conducted aboard the R/V ‘*Sarmiento de Gamboa*’ between March and April 2019, spanned ~9000 km along a latitudinal transect (48°S/26°N) in the Atlantic Ocean. Seawater was systematically collected at various depths in 27 stations to capture the structure of the DCM, which was delineated using a CTD profiler SBE 911plus, with samples taken at different depths: surface, above DCM, DCM (defined as the maximal fluorescence point), below DCM, and beyond the end of the fluorescence signal. Additionally, at six stations, samples were collected at a higher resolution throughout the DCM (10 sampling depths). At two stations (Station 10 and Station 16), the DCM showed two chlorophyll maxima, and samples were collected at each chlorophyll peak and in between. All samples underwent prefiltration through a 200-μm mesh to remove large plankton. Flow cytometry was used to assess the abundance of cyanobacteria, photosynthetic pico- and nanoeukaryotes, and heterotrophic prokaryotes with high and low nucleic acid content (HNA and LNA), as described in Gasol and Morán [35]. The concentration of inorganic nutrients, specifically nitrate, nitrite, phosphate, and silicate, was determined following the procedures outlined in González-Vega *et al.* [36]. Chlorophyll *a* (Chl *a*) and bacteriochlorophyll *a* (BChl *a*) concentrations were measured using high-performance liquid chromatography, and AAP cell abundances were estimated with epifluorescence microscopy (for details, see Gazulla *et al.* [20]). The mixed layer depths (MLD) were calculated from the potential temperature profiles derived from CTD data. We computed the differences in potential temperature for intervals of 5 m depth, and the MLD was defined as the shallowest depth where the difference, for a 5 m interval, surpassed 0.3°C.

### DNA extraction, amplification, and sequencing of the *pufM* gene

Approximately 2 l of prefiltered seawater were sequentially filtered through 20-μm and 3-μm prefilters onto a 0.2-μm 47 mm polycarbonate filter. Samples were subsequently stored at −80°C until further processing. DNA was extracted from the 0.2 μm filter (0.2–3 μm fraction) using the phenol–chloroform protocol described in Massana *et al.* [37]. Partial amplification of the *pufM* gene (~180 bp fragment) was carried out in 12.5 μL reactions using primers pufM_uniF (GGNAAYYTNTWYTAYAAYCCNTTYCA) and pufM_UniR (YCCATNGTCCANCKCCARAA), from Yutin *et al.* [38]. The amplification followed the conditions detailed in Gazulla *et al.* [32]. In brief, PCR conditions were an initial denaturation step at 95°C for 5 min, 35 cycles at 95°C (30 s), 48°C (45 s), 72°C (45 s), and a final elongation step at 72°C for 7 min. DNA sequencing was conducted on an Illumina MiSeq sequencer by AllGenetics & Biology SL (www.allgenetics.eu). Noteworthy, certain samples, particularly some from the DCM and below the DCM, could not be successfully amplified, likely due to the low abundance of AAP bacteria [20]. As a result, we were able to obtain high-quality sequencing results from 84 samples.

### Amplicon sequence variants generation and taxonomic assignation

Primers were removed with cutadapt v3.4 [39] and DADA2 v1.26 [40] was used to obtain amplicon sequence variants (ASVs) and eliminate chimeras. Taxonomy assignment of ASVs was performed using the Evolutionary Placement Algorithm v0.3.5 [41] against a *pufM* phylogenetic tree. The reference database to build the tree comprised full-length *pufM* gene sequences or long fragments (>600 nucleotides) retrieved from Genome Taxonomy Database (GTDB) and metagenomic datasets such as the *Tara* Oceans [10, 42], the Malaspina Expedition [43], the Global Ocean Survey [44], and the Blanes Bay Microbial Observatory [45]. Additionally, we included *pufM* gene sequences from metagenome-assembled genomes (MAGs) [33] and single-amplified genomes (SAGs) [34] affiliated to the newly described *Ca.* Luxescamonaceae family. The phylogenetic tree was constructed with RAxML-ng [46] and visualized using iTOL [47]. We defined a total of 13 different taxonomic groups based on the initial classification proposed by Yutin *et al.* [44] and encompassing different orders from the Gamma- and Alphaproteobacteria. Sequences that could not be further assigned were collectively grouped as ‘Others’.

### Data analyses

All analyses were conducted in R v4.3.3 (R Core Team 2024) [48]. The resulting ASV table underwent rarefaction down to 6576 reads using the ‘vegan’ package [49]. Diversity indices (Chao1 and evenness) were estimated using the ‘vegan’ package. To study differences across the vertical and horizontal scale, samples were categorized according to their position along the DCM profile and their corresponding Longhurst provinces [50]. Differences between groups were assessed by analyses of variance with the aov() function in the R Stats Package ‘stats,’ (version 3.6.2) and the post hoc Tukey’s ‘Honest Significant Difference’ method with function TukeyHDS() from the same package. Correlation between abundance changes in various taxonomic groups and environmental variables was estimated with the rcorr() function (‘Hmisc’ package [51]), applying a Bonferroni correction. Relative abundances of taxonomic groups were transformed using the centered log-ratio (CLR) transformation to account for the compositional nature of the data. Pearson correlations between groups were then computed on the CLR-transformed values. For this analysis, we included only taxonomic groups with a mean relative abundance above 3%. Ordination tests were based on nonmetric multidimensional scaling (NMDS) analysis using Bray–Curtis distances, and calculated with the vegdist() function (‘vegan’ package). The envfit() function (‘vegan’ package) was used to fit environmental vectors onto the ordination space. To test for significant clustering based on Longhurst provinces or the position along the DCM profile, we used the ‘envfit’ analysis and a PERMANOVA test using the adonis2() function, also in the ‘vegan’ package. Vertical connectivity was analyzed by classifying each ASV into four categories: ‘surface’, ‘above-DCM’, ‘DCM’, and ‘below-DCM’, according to the depth where it was first found, starting from the surface and going downward. Computing analyses were run at the MARBITS bioinformatics platform at the Institut de Ciències del Mar (ICM-CSIC) and at the Picasso Supercomputer at the Supercomputing and Bioinformatics Center of the University of Málaga.

## Results and discussion

### Oceanographic context

**Figure 1:**
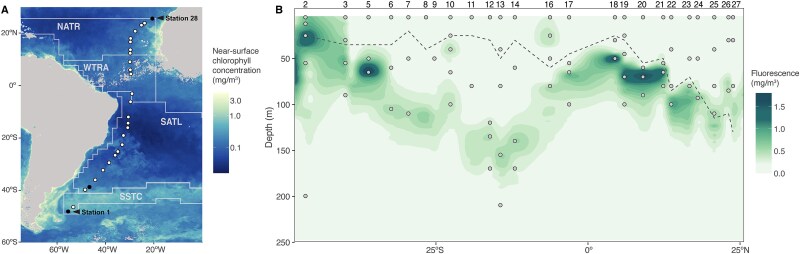
Chlorophyll distribution across the sampling transect. (A) Near-surface chlorophyll *a* concentrations and station positions along the Poseidon Expedition transect in the South and Central Atlantic (March 2019). Chlorophyll *a* (mg·m^−3^) values were obtained from SNPP-VIIRS data (https://oceancolor.gsfc.nasa.gov/l3/. White dots indicate stations whose DNA was sequenced in this study, whereas black dots mark stations where no DNA was sampled. The transect covered four Longhurst provinces: SSTC, South Subtropical Convergence zone; SATL, South Atlantic gyre; WTRA, Western tropical Atlantic; NATR, North Atlantic Tropical gyre. (B) Sectional distribution of *in situ* chlorophyll fluorescence measured by the CTD along the transect. Numbers above the panel indicate the station number. Dots indicate the sampling points. The dashed line represents the mixed layer depth at each station. Note that Station 15 does not exist. Figure adapted from Gazulla *et al.* [20].

The Poseidon Expedition went from south to north across four Longhurst oceanographic provinces [50] (Fig. 1A) in the South and Central Atlantic Ocean. It started in an area strongly influenced by the Southern Ocean; Station 2, located in the South Subtropical Convergence Province (SSTC), was characterized by low temperatures and salinity, with the DCM located at ~12 m deep (Fig. 1B). Moving northward, the transect entered the South Atlantic oligotrophic gyre (South Atlantic Gyre province, SATL), where both temperature and salinity increased (stations 3–14). This area was characterized by low surface chlorophyll *a* (Chl *a*), deeper DCMs, and low nutrient concentrations. Further north (Western Tropical Atlantic Province, WTRA), closer to the equator (stations 16–19), DCMs became progressively shallower, with increasing Chl *a* and nutrient concentrations due to upwelling. Beyond the equator, temperatures remained stable while salinity increased as the transect approached the North Atlantic oligotrophic gyre (North Atlantic Tropical Gyre, NATR). This final section was characterized by a strong vertical mixing and average mixed layer depth (MLD) descending up to 130 m deep (stations 22–27, mean 101.71 m ± 21.15 m, Fig. 1B). More details on the oceanographic conditions can be found in Gazulla *et al.* [20].

### Aerobic anoxygenic phototrophic bacterial diversity and community structure are shaped following the deep chlorophyll maximum profile

**Figure 2:**
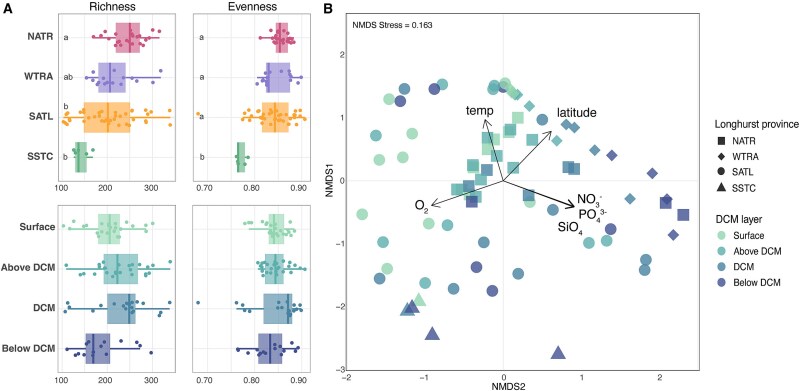
Alpha and beta diversity of AAP bacteria in the Atlantic Ocean. (A) Alpha diversity measured across latitude (horizontal scale, top panels) and vertically through the DCM structure (bottom panels). Richness was measured using the Chao1 index. Different letters indicate significant differences among groups (Tukey’s test, *P* < .05). Top panels include samples from the four DCM layers in each region. (B) Nonmetrical multidimensional (nMDS) plot based on the Bray–Curtis dissimilarities of AAP community composition. Samples from distinct DCM layers and Longhurst provinces can be differentiated. The ‘envfit’ analysis identified temperature (temp), oxygen (O_2_), nitrate (NO_3_^−^), phosphate (PO_4_^3−^), silicate (SiO_4_), and latitude as the main variables that explained the largest fraction of community variance (*R*^2^ >0.3, *P* = .001). SSTC, South Subtropical Convergence zone; SATL, South Atlantic gyre; WTRA, Western tropical Atlantic; NATR, North Atlantic Tropical gyre.

We identified a total of 3350 ASVs of the *pufM* gene in the 84 analyzed samples. Chao1 richness values varied between 103 and 339 (mean 214 ± 58), a fair representation of the diversity, as seen by the rarefaction curves (Fig. S1), yet surpassing observations in the same area during the Malaspina Expedition [28] or those in other oceanic sites (e.g. [26, 27, 45]). The high richness values could be explained by the use of primers with broader phylogenetic coverage than those previously used, as well as by the inclusion of samples from deeper layers of the euphotic zone. In this regard, a total of 1676 ASVs (49%) were exclusively present below the surface. Previous studies focused solely on the surface, ignoring the ocean vertical dimension, and thus missing a high fraction of AAP diversity. Our data show that in many stations, richness reached its maximum where Chl *a* concentration was highest (Fig. 2A, Fig. S2A and B, Spearman correlation of Chl *a* and Chao1 in WTRA; *n* = 16, *R* = 0.82, *P* < .01; Spearman correlation of Chl *a* and Chao1 in SATL; *n* = 39, *R* = 0.35, *P* < .05). This pattern was particularly evident at stations with the highest Chl *a* concentrations (Fig. S2B). Interestingly, the two stations with double DCM (Station 10 and Station 16) had different patterns (Fig. S3) and richness reached its maximum at the shallow DCM (45 m) in Station 10 and at the deeper DCM (80 m) in Station 16. Previous studies have also shown that bacterioplankton diversity increases from the surface toward deeper waters in the epipelagic [14, 15]. However, it seems that, rather than a gradient of richness with depth, richness of AAP communities follows the distribution of Chl *a,* regardless of the DCM structure. This trend contrasts with previous studies that reported the highest diversity values in areas with low Chl *a* concentrations [28, 52] and low inorganic nutrient concentrations [31, 53]. In this study, evenness values were generally high (mean 0.84 ± 0.04, Fig. 2A) and similar across the vertical and latitudinal gradient, being only remarkably low in samples from Station 2 in the SSTC province (Tukey’s test, *P* < .001, Fig. 2A). Stations from the NATR province, with a deeper mixed layer, harbored assemblages with high diversity (*n* = 84, Pearson corr. between MLD and Chao1, *R* = 0.39, *P* < .001, Pearson corr. between MLD and evenness, *R* = 0.30, *P* < .05; Fig. S4A and B) and homogeneous composition across the DCM profile, as seen by the low Bray–Curtis values of these stations (Fig. S4C). Finally, the decline of diversity that we observed with latitude aligns well with the global diversity trends observed previously for the whole prokaryotic plankton, where Shannon sharply decreased at high latitudes (above 40°), mainly driven by decreasing ocean temperatures [54].

To explore the biogeographical patterns of AAP communities across the vertical and latitudinal dimensions, we computed an NMDS coupled with an ‘envfit’ analysis, to discern the environmental variables influencing the distribution of assemblages (Fig. 2B). Oxygen (*R*^2^ = 0.53, *P* = .001), temperature (*R*^2^ = 0.53, *P* = .001), latitude (*R*^2^ = 0.45, *P* = .001), and nutrients (NO_3_^−^, *R*^2^ = 0.38, *P* = .001; SiO_4_, *R* = 0.38, *P* = .001; PO_4_^3−^, *R* = 0.38, *P* = .001) emerged as the main drivers (Fig. 2B) but others, such as salinity, fluorescence, and depth, also showed significant association to the ordination of samples (Table S1). Temperature, salinity, and the trophic status have also been defined as key variables influencing the diversity and composition of AAP communities in the surface ocean [26, 27, 44]. A study based on the surface global ocean showed that AAP communities are subjected to strong selection processes even when there are small changes in the environmental conditions [28]. The Poseidon Expedition sampling covered four Longhurst provinces, each characterized by a unique combination of environmental variability (Fig. S5) that may promote the selection of specific taxa within provinces. Indeed, we found a separation of samples according to their Longhurst province (‘envfit’ analysis: *R*^2^ = 0.32, *P* < .001; PERMANOVA: *R*^2^ = 0.10, *P* = .001, Fig. S6). This biogeographic classification has proven to be effective in explaining the spatial structure of various microbial communities [55–59], including those of AAP bacteria ([28] and this study). It is widely recognized that the environmental variability associated with depth is a major driver of changes in the composition of prokaryotic and eukaryotic communities [8–10, 43, 60–62], but in contrast, the effects of that vertical component in the distribution of AAP communities have barely been explored. To illustrate the vertical variability in our dataset, we classified the samples by their position along the DCM profile (surface, above, at, and below the DCM; Fig. 2A) rather than their depth, in order to better capture structure patterns linked to the DCM. We observed an ordination based on these layers in regions at and north of the equator (WTRA PERMANOVA: *R*^2^ = 0.53, *P* = .001; NATR PERMANOVA: *R*^2^ = 0.31, *P* = .003), indicating that community structure in these areas is strongly influenced by their position along the DCM profile. In the equatorial region, this pattern was associated with a marked stratification, characterized by intense chlorophyll maxima and high nutrient concentrations below the DCM. In contrast, stations in the NATR region were well mixed and nutrient-depleted. The vertical structuring of communities along the DCM gradient was evident in both regions, suggesting that this pattern emerges across distinct oceanographic conditions. Clear deviations from this ordination were observed in several SATL stations, including Station 10 (second DCM, at 100 m), Station 12 (below DCM, 170 m), Station 13 (below DCM, 210 m), and Station 14 (below DCM, 170 m), which were located in the oligotrophic gyre and characterized by deeper and weaker DCMs (Fig. 1B and Fig. 2B). A previous study based on *pufM* clone libraries in the Mediterranean Sea showed that populations above the DCM were more similar to each other than to those below the DCM [31]. More broadly, the DCM structure influences bacterioplankton community composition, with distinct assemblages associated with specific niches within and below this layer [14, 15]. At the same time, high vertical connectivity in the open ocean promotes the dominance of surface-associated taxa throughout the epipelagic zone and extends to the meso- and bathypelagic ocean [11]. In contrast, our results show that a larger fraction of the AAP community consists of sequences exclusively found in the DCM or below the DCM (Fig. S7), suggesting that AAP bacteria display a strong vertical structuring across the epipelagic area. Differences in bacterial communities across the epipelagic zone have been investigated mainly by differentiating between samples at the surface and the DCM [10, 11, 13, 54, 56, 58] reporting a general increase in bacterial diversity at the DCM relative to surface waters. In this study, we have significantly expanded the vertical resolution by considering different layers along the DCM structure and our results show that, besides the influence that can be attributed to the Longhurst provinces, the DCM structure influences the composition of AAP communities in the epipelagic zone. This association between AAP bacteria and the DCM profile likely reflects their photoheterotrophic lifestyle, as they rely on both light and dissolved organic matter, and supports the idea of potential ecological coupling between AAP bacteria and phytoplankton [20].

### *Ca.* Luxescamonaceae is the dominant AAP lineage in the euphotic ocean

**Figure 3:**
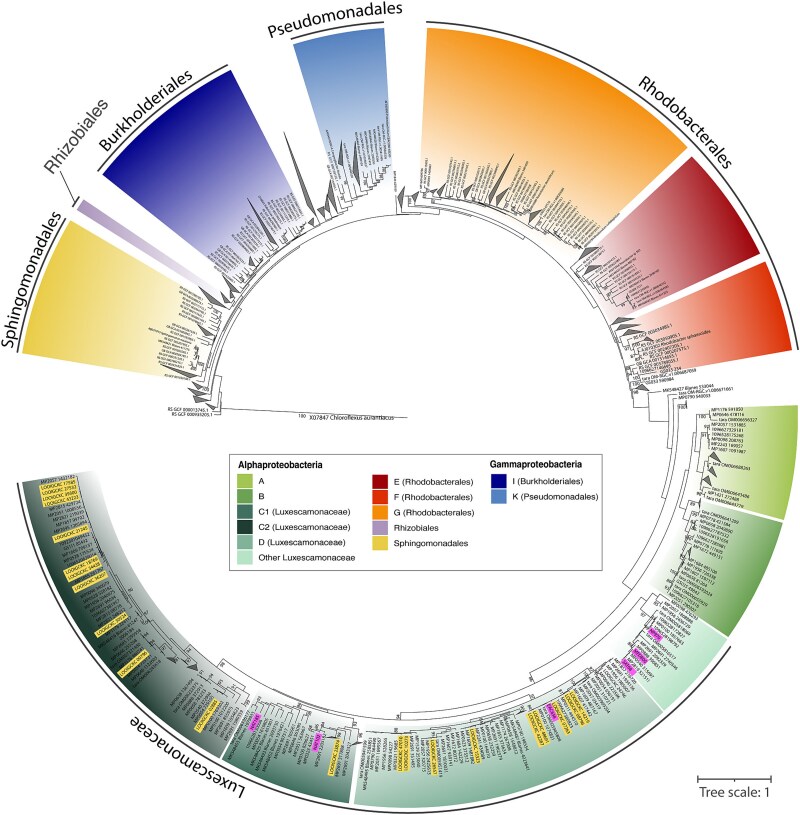
Phylogenetic tree of *pufM* gene sequences. Purple labels indicate *pufM* sequences related to *Ca.* Luxescamonaceae from the MAGs described in Graham *et al.* [33]. Yellow labels indicate *pufM* sequences related to *Ca.* Luxescamonaceae from SAGs identified by Pachiadaki *et al.* [34]. Only bootstrap support values above 80 are displayed.

We assigned the *pufM* gene sequences to specific taxonomic groups based on their placement in a phylogenetic tree (Fig. 3). Our classification used the phylogroups defined by Yutin *et al.* [44] complemented with the GTDB taxonomy, considering different orders of the classes Alphaproteobacteria (Sphingomonadales, Rhodobacterales, and Rhizobiales) and Gammaproteobacteria (Pseudomonadales and Burkholderiales). The 3350 *pufM* sequence variants were classified into 13 distinct taxonomic groups. Five of these groups—phylogroups A, B, C1, C2, D, and ‘Other Luxescamonaceae’ (Fig. 3)—have very few cultured representatives and mainly contain *pufM* gene sequences extracted from metagenomes, MAGs and SAGs from various marine regions.

**Figure 4:**
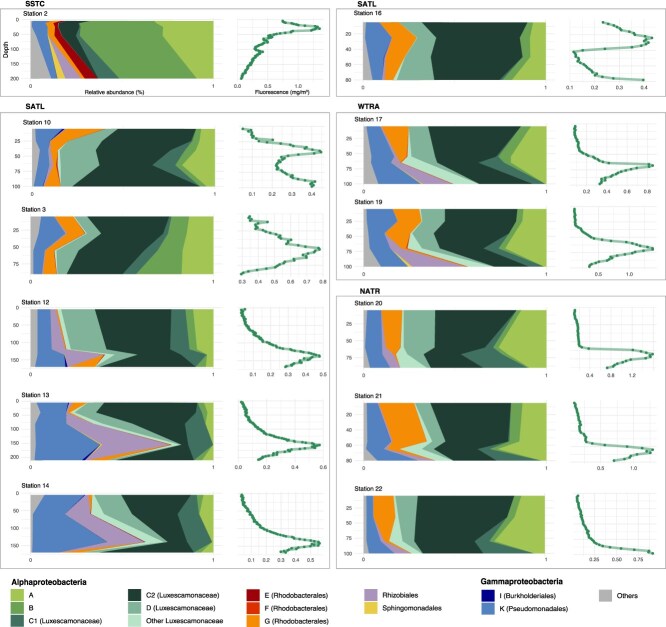
Taxonomy of AAP communities along the vertical profile. Taxonomic composition of AAP bacterial communities (left of each panel) and depth fluorescence variation (right of each panel) across depths in 12 stations along the Poseidon Expedition transect (only stations with sequencing data in more than three sampling points are shown). Stations from different Longhurst provinces are delimited within different boxes. SSTC, South Subtropical Convergence zone; SATL, South Atlantic gyre; WTRA, Western tropical Atlantic; NATR, North Atlantic Tropical gyre.

Phylogroup C2 consistently dominated across most stations and depths (mean relative abundance ± SD 37.68% ± 1.44), followed by phylogroup A (13.51% ± 7.80), phylogroup K within the Halieaceae family of the Pseudomonadales order (10.06% ± 6.89), phylogroup D (9.79% ± 6.13), phylogroup B (8.41% ± 10.83), and phylogroup G within the Rhodobacterales (6.76% ± 5.69) (Fig. 4, Table S2, Fig. S8). Other taxonomic groups exhibited mean relative abundances below 3%. Overall, the taxonomic composition of AAP communities was strongly structured by the DCM profile rather than by depth (Fig. 4). Clear shifts in community composition occurred across regions with contrasting oceanographic conditions and different DCM depths. For instance, in stations 12, 13, and 14, located within the oligotrophic gyre where the DCM reached ~150 m, community changes at the DCM were marked by increases in a few ASVs from phylogroup K (Pseudomonadales) and Rhizobiales. In contrast, stations in the north of the equator (stations 19–22, Fig. 4), with shallower and more intense chlorophyll peaks (>1 mg·m^−3^), exhibited shifts in taxonomic composition, with an increase in the abundance of phylogroups A and B, ‘Other Luxescamonaceae’, and a decrease in the relative abundance of phylogroup G (Rhodobacterales). In addition, AAP assemblages varied across oceanic regions. For example, Station 2, influenced by Southern Ocean conditions, harbored several taxa from the Sphingomonadales and phylogroup E (Rhodobacterales), which were nearly absent elsewhere (Fig. 4).

**Figure 5:**
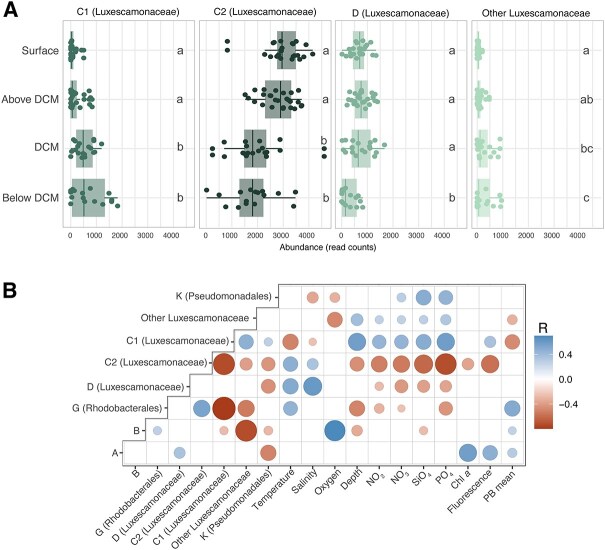
Luxescamonaceae distribution and environmental correlations. (A) Distribution of abundance of the four subclusters classified as *Ca.* Luxescamonaceae, along the DCM profile. Read counts represent values after rarefaction. Different letters indicate significant differences among groups (Tukey’s test, *P* < .05). (B) Pearson correlation coefficients between the relative abundance of the most prevalent taxonomic groups (mean relative abundance above 3%) and environmental variables. The plot only displays statistically significant (*P* < .05) correlations (R) after Bonferroni correction. Chl *a*, chlorophyll *a* concentration; PB mean, Mean values of heterotrophic bacterial production.

Contrary to previous assumptions, our results challenge the prevailing view that Pseudomonadales (phylogroup K) and Rhodobacterales (particularly phylogroup G) dominate AAP assemblages in the open ocean [26, 28, 31, 52]. The taxonomic composition observed here is similar to that reported in Yutin *et al.* [44], which was based solely on metagenomic data. In fact, our choice of primers follows Gazulla *et al.* [32], in which we demonstrated that traditionally employed primers have multiple mismatches for phylogroups A, B, C, and D, leading to their underestimation. By using primers with higher phylogenetic coverage, we revealed that, indeed, these groups constitute a substantial fraction of AAP communities along both the vertical and horizontal continuum. Interestingly, little ecological or physiological information exists for these groups, as no representative isolates are currently available. The phylogenetic analyses showed that phylogroups C and D clustered with sequences from the family *Ca.* Luxescamonaceae (bootstrap support = 0.86, Fig. 3). First reported from the *Tara* Oceans metagenomic dataset [33], the clade *Ca.* Luxescamonaceae was later described in SAGs from the same expedition [34]. The study of these genomes revealed not only genomic potential for anoxygenic phototrophy but also for carbon fixation via the Calvin–Benson–Bassham cycle, using ribulose-1,5-bisphosphate carboxylase (RuBisCO) form IC/D. Both studies place this clade within the Alphaproteobacteria, and a recent study defines them as the LUX cluster within the *Roseobacter* group [63]. In addition, our phylogenetic tree shows that this cluster could be further divided into four subclusters: two robust subclusters (BS > 95), phylogroup D and part of phylogroup C (cluster C2), and another two subclusters with lower confidence level that represent phylogroup C (cluster C1, BS = 0.35) and other sequences that could not be assigned to any of these phylogroups, and that we named ‘Other Luxescamonaceae’ (BS = 0.68, Fig. 3A). A comparison of our phylogenetic tree with that of Liu *et al.* [63] suggests that phylogroup C2 may be related to subcluster LUX-A, while phylogroup D may be related to subcluster LUX-I, both comprising sequences from the Atlantic Ocean. Previous studies analyzing the phylogeny of *pufM* gene sequences show very similar phylogenetic clustering for phylogroups C and D [28, 45]. In our study, a total of 1579 ASVs (47.13% of the total number of ASVs) were assigned to the clusters within the *Ca.* Luxescamonaceae family, representing more than half of the total AAP relative abundance (54.86% ± 14.53, Table S2). Among them, we found contrasting vertical distribution patterns. Luxescamonaceae C2 was more abundant at the surface and decreased progressively through the DCM profile, whereas Luxescamonaceae C1 peaked below the DCM (Fig. 5A). Clade C1 thrived in deeper, nutrient-rich and colder waters while clades C2 and D correlate with the higher salinity and warmer temperatures found in surface waters of the Atlantic Ocean (Fig. 5B). Based on SAGs and MAGs recovered from tropical to temperate waters across major oceans and adjacent seas, the LUX cluster comprises genomes showing significant differences in genome features such as genome size, G + C content, and coding density [63]. These genomic differences, together with the observed variation in ecological niches observed in our study, suggest that *Ca.* Luxescamonaceae is not a single cohesive ecological group but rather contains various ecotypes adapted to the distinct conditions within the euphotic ocean. While phylogroups C and D are known to be related to *Ca.* Luxescamonaceae, much less is known about phylogroups A and B. Initially identified in oligotrophic regions of the Atlantic and Pacific oceans [44], they were recently found to dominate AAP communities in the Mediterranean Sea, particularly during early spring and winter [64]. In our study, phylogroup A consistently peaked at the DCM and showed a positive correlation with Chl *a* concentration and fluorescence (Pearson corr. with Chl *a R* = 0.56, *P* < .001; fluorescence *R* = 0.57, *P* < .001, Fig. 3C). Phylogroup B was generally scarce across the dataset, except at Station 2, where it accounted for ~40% of the community from the surface down to 200 m depth. This station, located in the SSTC region and highly influenced by its proximity to the Southern Ocean, had a clearly different AAP community, as revealed by ordination analyses (Fig. 2B and Fig. S6). These observations suggest that phylogroup B may be better adapted to high-latitude, cold-water environments.

Although typically studied collectively as a cohesive microbial guild, AAP bacteria comprise very diverse metabolisms and life strategies, ranging from generalists to specialists that thrive on different ranges of carbon sources [64], and with distinct habitat preferences [27, 50, 64, 65]. By integrating vertical patterns across the subtropical and tropical ocean, this study provides new insight into the ecological preferences of AAPs and, for the first time, describes the ecological distribution of the abundant *Ca.* Luxescamonaceae family within these communities. It remains unknown whether the *pufM* gene sequences recovered in our study originate from genomes with carbon fixation potential, and if so, whether these genes are functionally active. AAP bacteria are traditionally defined as a functional group of strict photoheterotrophs [66, 67], and the potential for carbon fixation within the Luxescamonaceae clade remains to be experimentally demonstrated. If confirmed, this would challenge the current functional definition of AAP bacteria and may require its reconsideration, either by broadening the definition of AAP bacteria to include metabolically flexible phototrophs or establishing a distinct functional category. In any case, given the high abundance of Luxescamonaceae throughout the Atlantic Ocean, such a finding would also require a reevaluation of the ecological roles traditionally attributed to AAP bacteria in aquatic systems.

## Conclusions

This study shows for the first time that AAP bacterial diversity and community structure closely follow the DCM, with richness peaking at or just above the DCM and correlating with chlorophyll *a* concentrations. Temperature, oxygen, latitude, and nutrient concentrations are the primary environmental drivers shaping AAP communities, with salinity and vertical position within the DCM also contributing. Biogeographic patterns are further structured by Longhurst provinces, highlighting the role of environmental selection. Our results reveal that previously underestimated uncultured phylogroups (A, B, C, and D) constitute a substantial fraction of AAP communities, likely overlooked due to primer biases. Remarkably, *Ca.* Luxescamonaceae dominates the Atlantic Ocean euphotic zone, comprising multiple subclusters with distinct vertical distributions, suggesting different ecotypes adapted to varying environmental conditions. Overall, these findings underscore the ecological diversity and environmental specialization of AAP bacteria, while highlighting important knowledge gaps that could affect our understanding of their contribution to marine phototrophic processes.

## Supplementary Material

ycag076_Supplemental_Files

## Data Availability

The code of the analyses performed can be found at https://github.com/crgazulla/AAPs_DCMprofile. *pufM* amplicon sequences have been deposited in the NCBI Sequence Read Archive (SRA) under BioProject ID PRJNA1049819.

## References

[1] Cullen JJ . Subsurface chlorophyll maximum layers: enduring enigma or mystery solved? *Ann Rev Mar Sci* 2015;7:207–39. 10.1146/annurev-marine-010213-135111 25251268

[2] Cullen JJ . The deep chlorophyll maximum: comparing vertical profiles of chlorophyll *a*. *Can J Fish Aquat Sci* 1982;39:791–803. 10.1139/f82-108

[3] Estrada M, Marrase C, Latasa M. et al. Variability of deep chlorophyll maximum characteristics in the northwestern Mediterranean. *Mar Ecol Prog Ser* 1993;92:289–300. 10.3354/meps092289

[4] Sharples J, Moore CM, Rippeth TP. et al. Phytoplankton distribution and survival in the thermocline. *Limnol Oceanogr* 2001;46:486–96. 10.4319/lo.2001.46.3.0486

[5] Lorenzo LM, Figueiras FG, Tilstone GH. et al. Photosynthesis and light regime in the Azores front region during summer: are light-saturated computations of primary production sufficient? *Deep Res Part I Oceanogr Res Pap* 2004;51:1229–44. 10.1016/j.dsr.2004.01.010

[6] Weston K, Fernand L, Mills DK. et al. Primary production in the deep chlorophyll maximum of the Central North Sea. *J Plankton Res* 2005;27:909–22. 10.1093/plankt/fbi064

[7] Marañón E, Wambeke FV, Uitz J. et al. Deep maxima of phytoplankton biomass, primary production and bacterial production in the Mediterranean Sea. *Biogeosciences* 2021;18:1749–67. 10.5194/bg-18-1749-2021

[8] Cabello AM, Latasa M, Forn I. et al. Vertical distribution of major photosynthetic picoeukaryotic groups in stratified marine waters. *Environ Microbiol* 2016;18:1578–90. 10.1111/1462-2920.13285 26971724

[9] Latasa M, Cabello AM, Morán XAG. et al. Distribution of phytoplankton groups within the deep chlorophyll maximum. *Limnol Oceanogr* 2017;62:665–85. 10.1002/lno.10452

[10] Sunagawa S, Coelho LP, Chaffron S. et al. Structure and function of the global ocean microbiome. *Science* 2015;348:1–10. 10.1126/science.1261359 25999513

[11] Mestre M, Ruiz-González C, Logares R. et al. Sinking particles promote vertical connectivity in the ocean microbiome. *Proc Natl Acad Sci U S A* 2018;115:E6799–807. 10.1073/pnas.1802470115 29967136 PMC6055141

[12] Ruiz-González C, Logares R, Sebastián M. et al. Higher contribution of globally rare bacterial taxa reflects environmental transitions across the surface ocean. *Mol Ecol* 2019;28:1930–45. 10.1111/mec.15026 30663830

[13] Walsh EA, Smith DC, Sogin ML. et al. Bacterial and archaeal biogeography of the deep chlorophyll maximum in the South Pacific gyre. *Aquat Microb Ecol* 2015;75:1–13. 10.3354/ame01746

[14] Haro-Moreno JM, López-Pérez M, de la Torre JM. et al. Fine metagenomic profile of the Mediterranean stratified and mixed water columns revealed by assembly and recruitment. *Microbiome* 2018;6:1–19. 10.1186/s40168-018-0513-5 29991350 PMC6040077

[15] Marín-Vindas C . Patterns of connectivity in marine microbial communities. *Doctoral Thesis, Universitat Politècnica Catalunya* 2023.

[16] Gómez-Consarnau L, González JM, Coll-Lladó M. et al. Light stimulates growth of proteorhodopsin-containing marine Flavobacteria. *Nature* 2007;445:210–3. 10.1038/nature05381 17215843

[17] Gómez-Consarnau L, Raven JA, Levine NM. et al. Microbial rhodopsins are major contributors to the solar energy captured in the sea. *Sci Adv* 2019;5:1–8. 10.1126/sciadv.aaw8855 PMC6685716 31457093

[18] Gómez-Consarnau L, Akram N, Lindell K. et al. Proteorhodopsin phototrophy promotes survival of marine bacteria during starvation. *PLoS Biol* 2010;8:2–11. 10.1371/journal.pbio.1000358 PMC2860489 20436956

[19] Hojerová E, Mašin M, Brunet C. et al. Distribution and growth of aerobic anoxygenic phototrophs in the Mediterranean Sea. *Environ Microbiol* 2011;13:2717–25. 10.1111/j.1462-2920.2011.02540.x 21883792

[20] Gazulla CR, Koblížek M, Mercado JM. et al. Aerobic anoxygenic phototrophic bacteria correlate with picophytoplankton across the Atlantic Ocean but show unique vertical bioenergetics. *Limnol Oceanogr* 2024;69:2503–15. 10.1002/lno.12682

[21] Fauteux L, Cottrell MT, Kirchman DL. et al. Patterns in abundance, cell size and pigment content of aerobic anoxygenic phototrophic bacteria along environmental gradients in northern lakes. *PloS One* 2015;10:e0124035. 10.1371/journal.pone.0124035 25927833 PMC4415779

[22] Cottrell MT, Mannino A, Kirchman DL. Aerobic anoxygenic phototrophic bacteria in the mid-Atlantic bight and the North Pacific gyre. *Appl Environ Microbiol* 2006;72:557–64. 10.1128/AEM.72.1.557-564.2006 16391092 PMC1352302

[23] Mašín M, Zdun A, Stoń-Egiert J. et al. Seasonal changes and diversity of aerobic anoxygenic phototrophs in the Baltic Sea. *Aquat Microb Ecol* 2006;45:247–54. 10.3354/ame045247

[24] Boeuf D, Cottrell MT, Kirchman DL. et al. Summer community structure of aerobic anoxygenic phototrophic bacteria in the western Arctic Ocean. *FEMS Microbiol Ecol* 2013;85:417–32. 10.1111/1574-6941.12130 23560623

[25] Lehours A, Jeanthon C. The hydrological context determines the beta-diversity of aerobic anoxygenic phototrophic bacteria in European Arctic seas but does not favor endemism. *Front Microbiol* 2015;6:1–9. 10.3389/fmicb.2015.00638 26191046 PMC4490794

[26] Bibiloni-Isaksson J, Seymour JR, Ingleton T. et al. Spatial and temporal variability of aerobic anoxygenic photoheterotrophic bacteria along the east coast of Australia. *Environ Microbiol* 2016;18:4485–500. 10.1111/1462-2920.13436 27376620

[27] Lehours AC, Enault F, Boeuf D. et al. Biogeographic patterns of aerobic anoxygenic phototrophic bacteria reveal an ecological consistency of phylogenetic clades in different oceanic biomes. *Sci Rep* 2018;8:1–10. 10.1038/s41598-018-22413-7 29515205 PMC5841314

[28] Gazulla CR, Auladell A, Ruiz-González C. et al. Global diversity and distribution of aerobic anoxygenic phototrophs in the tropical and subtropical oceans. *Environ Microbiol* 2022;24:2222–38. 10.1111/1462-2920.15835 35084095

[29] Sieracki ME, Gilg IC, Thier EC. et al. Distribution of planktonic aerobic anoxygenic photoheterotrophic bacteria in the Northwest Atlantic. *Limnol Oceanogr* 2006;51:38–46. 10.4319/lo.2006.51.1.0038

[30] Lami R, Cottrell MT, Ras J. et al. High abundances of aerobic anoxygenic photosynthetic bacteria in the South Pacific Ocean. *Appl Environ Microbiol* 2007;73:4198–205. 10.1128/AEM.02652-06 17496136 PMC1932784

[31] Lehours AC, Cottrell MT, Dahan O. et al. Summer distribution and diversity of aerobic anoxygenic phototrophic bacteria in the Mediterranean Sea in relation to environmental variables. *FEMS Microbiol Ecol* 2010;74:397–409. 10.1111/j.1574-6941.2010.00954.x 21039650

[32] Gazulla CR, Cabello AM, Sánchez P. et al. A metagenomic and amplicon sequencing combined approach reveals the best primers to study marine aerobic anoxygenic phototrophs. *Microb Ecol* 2023;86:2161–72. 10.1007/s00248-023-02220-y 37148309 PMC10497671

[33] Graham ED, Heidelberg JF, Tully BJ. Potential for primary productivity in a globally-distributed bacterial phototroph. *ISME J* 2018;12:1861–6. 10.1038/s41396-018-0091-3 29523891 PMC6018677

[34] Pachiadaki MG, Brown JM, Brown J. et al. Charting the complexity of the marine microbiome through single-cell genomics. *Cell* 2019;179:1623–1635.e11. 10.1016/j.cell.2019.11.017 31835036 PMC6919566

[35] Gasol JM, Morán XAG. Flow cytometric determination of microbial abundances and its use to obtain indices of community structure and relative activity. 2015. In: McGenity TJ, Timmis KN, Nogales B (eds) Hydrocarbon and Lipid Microbiology Protocols. Springer Protocols Handbooks. Berlin, Heidelberg: Springer. 10.1007/8623_2015_139

[36] González-Vega A, Arrieta JM, Santana-Casiano M. et al. Tagoro submarine volcano as a natural source of significant dissolved inorganic nutrients. 2023. In: González PJ (ed.), El Hierro Island, Active Volcanoes of the World. Cham: Springer. 10.1007/978-3-031-35135-8_9

[37] Massana R, Murray AE, Preston CM. Vertical distribution and phylogenetic characterization of marine planktonic archaea in the Santa Barbara Channel. *Appl Environ Microbiol* 1997;63:50–6. 10.1128/aem.63.1.50-56.1997 8979338 PMC168301

[38] Yutin N, Suzuki MT, Béjà O. Novel primers reveal wider diversity among marine aerobic anoxygenic phototrophs. *Appl Environ Microbiol* 2005;71:8958–62. 10.1128/AEM.71.12.8958-8962.2005 16332899 PMC1317425

[39] Martin M . Cutadapt removes adapter sequences from high-throughput sequencing reads. *EMBnet J* 2013;17:10. 10.14806/ej.17.1.200

[40] Callahan BJ, McMurdie PJ, Rosen MJ. et al. DADA2: high-resolution sample inference from Illumina amplicon data. *Nat Methods* 2016;13:581–3. 10.1038/nmeth.3869 27214047 PMC4927377

[41] Barbera P, Kozlov AM, Czech L. et al. EPA-ng: massively parallel evolutionary placement of genetic sequences. *Syst Biol* 2019;68:365–9. 10.1093/sysbio/syy054 30165689 PMC6368480

[42] Pesant S, Not F, Picheral M. et al. Open science resources for the discovery and analysis of Tara oceans data. *Sci Data* 2015;2:1–16. 10.1038/sdata.2015.23 PMC4443879 26029378

[43] Sánchez P, Coutinho FH, Sebastián M. et al. Marine picoplankton metagenomes and MAGs from eleven vertical profiles obtained by the Malaspina expedition. *Sci Data* 2024;11:154. 10.1038/s41597-024-02974-1 38302528 PMC10834958

[44] Yutin N, Suzuki MT, Teeling H. et al. Assessing diversity and biogeography of aerobic anoxygenic phototrophic bacteria in surface waters of the Atlantic and Pacific oceans using the Global Ocean sampling expedition metagenomes. *Environ Microbiol* 2007;9:1464–75. 10.1111/j.1462-2920.2007.01265.x 17504484

[45] Auladell A, Sánchez P, Sánchez O. et al. Long-term seasonal and interannual variability of marine aerobic anoxygenic photoheterotrophic bacteria. *ISME J* 2019;13:1975–87. 10.1038/s41396-019-0401-4 30914777 PMC6776013

[46] Kozlov AM, Darriba D, Flouri T. et al. RAxML-NG: a fast, scalable and user-friendly tool for maximum likelihood phylogenetic inference. *Bioinformatics* 2019;35:4453–5. 10.1093/bioinformatics/btz305 31070718 PMC6821337

[47] Letunic I, Bork P. Interactive tree of life (iTOL) v6: recent updates to the phylogenetic tree display and annotation tool. *Nucleic Acids Res* 2024;52:W78–82. 10.1093/nar/gkae268 38613393 PMC11223838

[48] R Core Team. R: A language and environment for statistical computing . R Foundation for Statistical Computing. Vienna: Austria, 2024, https://www.R-project.org/.

[49] Oksanen J, Simpson G, Blanchet F. et al. Vegan: community ecology package. *R package version* 2022;2:6–2 https://cran.r-project.org/package=vegan.

[50] Longhurst A, Sathyendranath S, Platt T. et al. An estimate of global primary production in the ocean from satellite radiometer data. *J Plankton Res* 1995;17:1245–71. 10.1093/plankt/17.6.1245

[51] F Harrell, C Dupont. Hmisc: Harrell Miscellaneous. 2023. Available at: https://cran.r-project.org/web/packages/Hmisc/index.html.

[52] Jiao N, Zhang Y, Zeng Y. et al. Distinct distribution pattern of abundance and diversity of aerobic anoxygenic phototrophic bacteria in the global ocean. Environ Microbiol 2007;9:3091–9. 10.1111/j.1462-2920.2007.01419.x 17991036

[53] Jeanthon C, Boeuf D, Dahan O. et al. Diversity of cultivated and metabolically active aerobic anoxygenic phototrophic bacteria along an oligotrophic gradient in the Mediterranean Sea. *Biogeosciences* 2011;8:1955–70. 10.5194/bg-8-1955-2011

[54] Ibarbalz FM, Henry N, Brandão CM. et al. Global trends in marine plankton diversity across kingdoms of life. *Cell* 2019;179:1084–1097.e21. 10.1016/j.cell.2019.10.008 31730851 PMC6912166

[55] Ruiz-González C, Mestre M, Estrada E. et al. Major imprint of surface plankton on deep ocean prokaryotic structure and activity. *Mol Ecol* 2020;29:1820–38. 10.1111/mec.15454 32323882

[56] Friedline CJ, Franklin RB, McCallister SL. et al. Bacterial assemblages of the eastern Atlantic Ocean reveal both vertical and latitudinal biogeographic signatures. *Biogeosciences* 2012;9:2177–93. 10.5194/bg-9-2177-2012

[57] Frank AH, Garcia JAL, Herndl GJ. et al. Connectivity between surface and deep waters determines prokaryotic diversity in the North Atlantic deep water. *Environ Microbiol* 2016;18:2052–63. 10.1111/1462-2920.13237 26914787 PMC4921061

[58] Milici M, Tomasch J, Wos-Oxley ML. et al. Bacterioplankton biogeography of the Atlantic Ocean: a case study of the distance-decay relationship. *Front Microbiol* 2016;7:1–15. 10.3389/fmicb.2016.00590 27199923 PMC4845060

[59] Logares R, Deutschmann IM, Junger PC. et al. Disentangling the mechanisms shaping the surface ocean microbiota. *Microbiome* 2020;2020:1–17. 10.1186/s40168-020-00827-8 PMC7171866 32312331

[60] Acinas SG, Antón J, Rodríguez-Valera F. Diversity of free-living and attached bacteria in offshore western Mediterranean waters as depicted by analysis of genes encoding 16S rRNA. *Appl Environ Microbiol* 1999;65:514–22. 10.1128/AEM.65.2.514-522.1999 9925576 PMC91055

[61] Salazar G, Cornejo-Castillo F, Benítez-Barrios V. et al. Global diversity and biogeography of deep-sea pelagic prokaryotes. *ISME J* 2016;10:596–608. 10.1038/ismej.2015.137 26251871 PMC4817678

[62] Junger PC, Sarmento H, Giner CR. et al. Global biogeography of the smallest plankton across ocean depths. *Sci Adv* 2023;9:1–15. 10.1126/sciadv.adg9763 PMC10631730 37939185

[63] Liu Y, Brinkhoff T, Simon M. Ecogenomics and functional biogeography of the *Roseobacter* group in the global oceans based on 653 MAGs and SAGs. *Microbiome* 2025;13:247. 10.1186/s40168-025-02259-8 41310780 PMC12661815

[64] Santos-Bruña JJ, Gazulla CR, Cabello AM. et al. Contrasting free-living and particle-associated photoheterotrophic bacterial communities across space and time in the Alboran Sea. Unpublished results. 10.1093/ismeco/ycag120 PMC13219738 42221915

[65] Villena-Alemany C, Tomaš AV, Mujakić I. et al. Lineage-specific phototrophy and lifestyle of coastal marine aerobic anoxygenic phototrophs. *Ocean Microbiol* 2025;1:1–5. 10.1186/s44375-025-00005-x

[66] Koblížek M . Ecology of aerobic anoxygenic phototrophs in aquatic environments. *FEMS Microbiol Rev* 2015;39:854–70. 10.1093/femsre/fuv032 26139241

[67] Yurkov V, Csotonyi JT. New light on aerobic anoxygenic phototrophs. In: Hunter CN, Dalda F, Thurnauer MC, Beatty JT. (eds) The Purple Phototrophic Bacteria. Advances in Photosynthesis and Respiration. 2009, 28. Dordrecht: Springer. 10.1007/978-1-4020-8815-5_3

