# Supplementary material for: Diversity and community structure of aerobic anoxygenic phototrophic bacteria are shaped by the deep chlorophyll maximum
Source: ISME Commun. 2026 Mar 26;6(1):ycag076. doi: 10.1093/ismeco/ycag076 (PMC13219746; doi:10.1093/ismeco/ycag076)
Supplement: ycag076_Supplemental_Files [file ycag076_supplemental_files.zip › Gazulla2026_Supp_Information_ycag076.docx]

Supplementary Information

for the manuscript

“Diversity and community structure of aerobic anoxygenic phototrophic bacteria are shaped by the deep chlorophyll maximum”

Fig. S1: Rarefaction curves

Fig. S2: Correlation of chlorophyll *a* and richness

Fig. S3: Stations with double DCM

Fig. S4: Correlation of the mean mixed layer depth and diversity

Fig. S5: Environmental variability at the Longhurst provinces

Fig. S6: Non-metrical multidimensional (nMDS)

Fig. S7: Vertical connectivity of AAP bacteria

Fig. S8: Taxonomic composition of samples

Table S1: *Envfit* analysis result

Table S2: Mean relative abundance and standard deviation of taxonomic groups


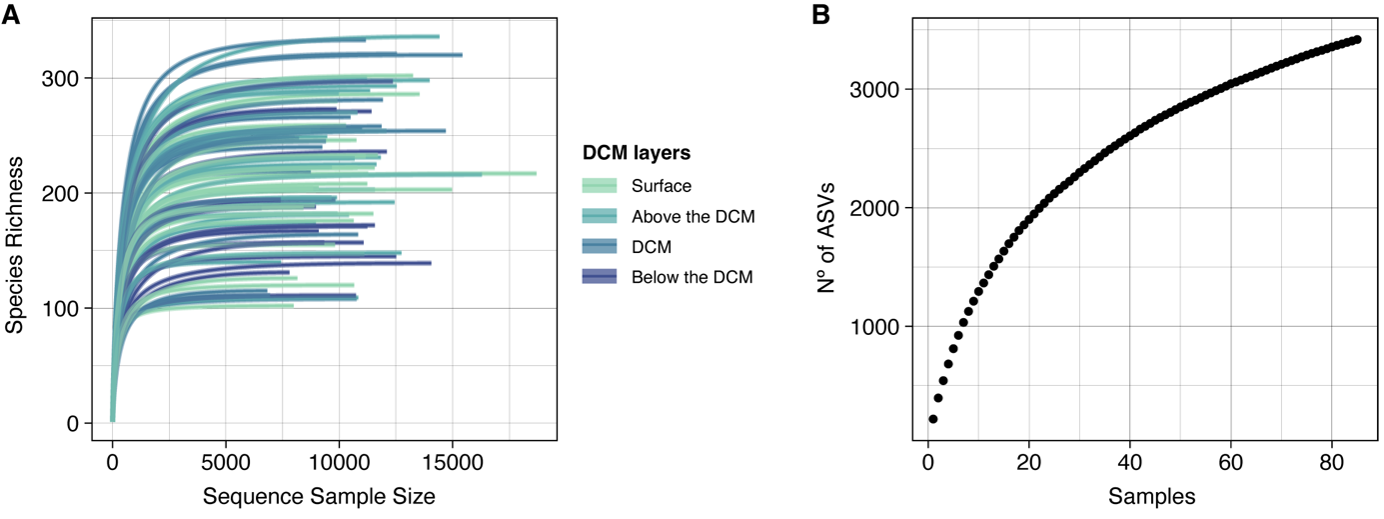


**Figure S1. A)** Rarefaction curves for each sample colour-coded by layers along the deep chlorophyll maximum (DCM) structure. **B)** Sample-based rarefaction curve, considering all samples collectively.


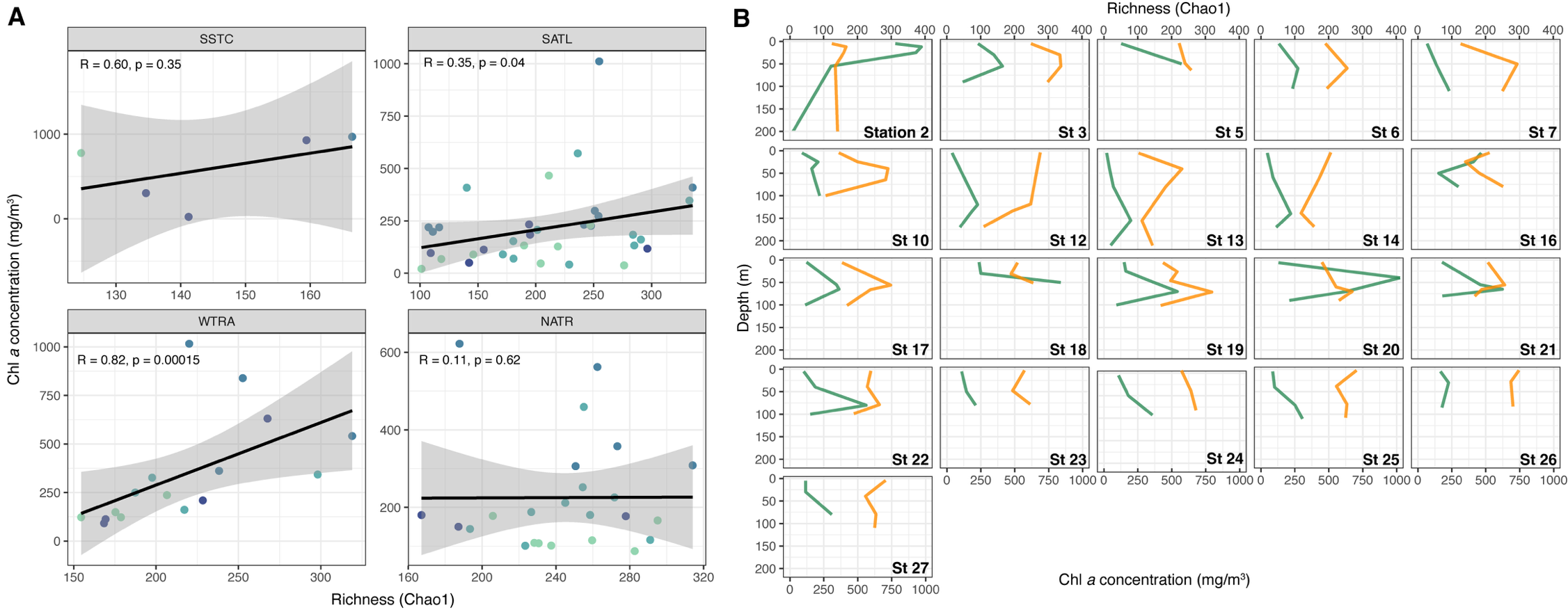


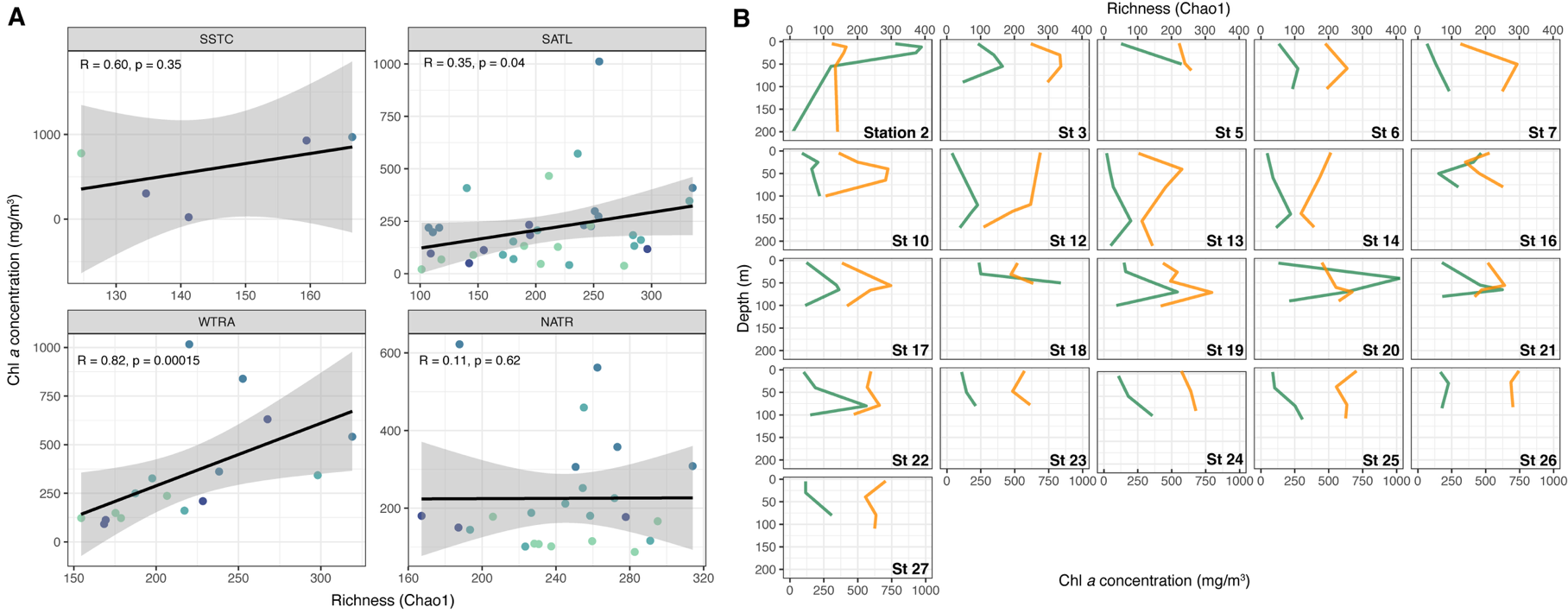


**Figure S2. A)** Correlation between richness (Chao1) and chlorophyll *a* concentration (Chl *a*) at each Longhurst province. Spearman correlation coefficient (R) and significance (p) is estimated for each Longhurst province and shown in each panel. **B)** Depth profile of the chlorophyll *a* and richness (Chao1) variation at each station. Only stations with three or more simples are shown.

**Figure S3.** Depth fluorescence variation (left panels) depicted by the CTD probe and Chao1 values (right panels) at stations 10 and 16, characterized by a double deep chlorophyll maximum structure.


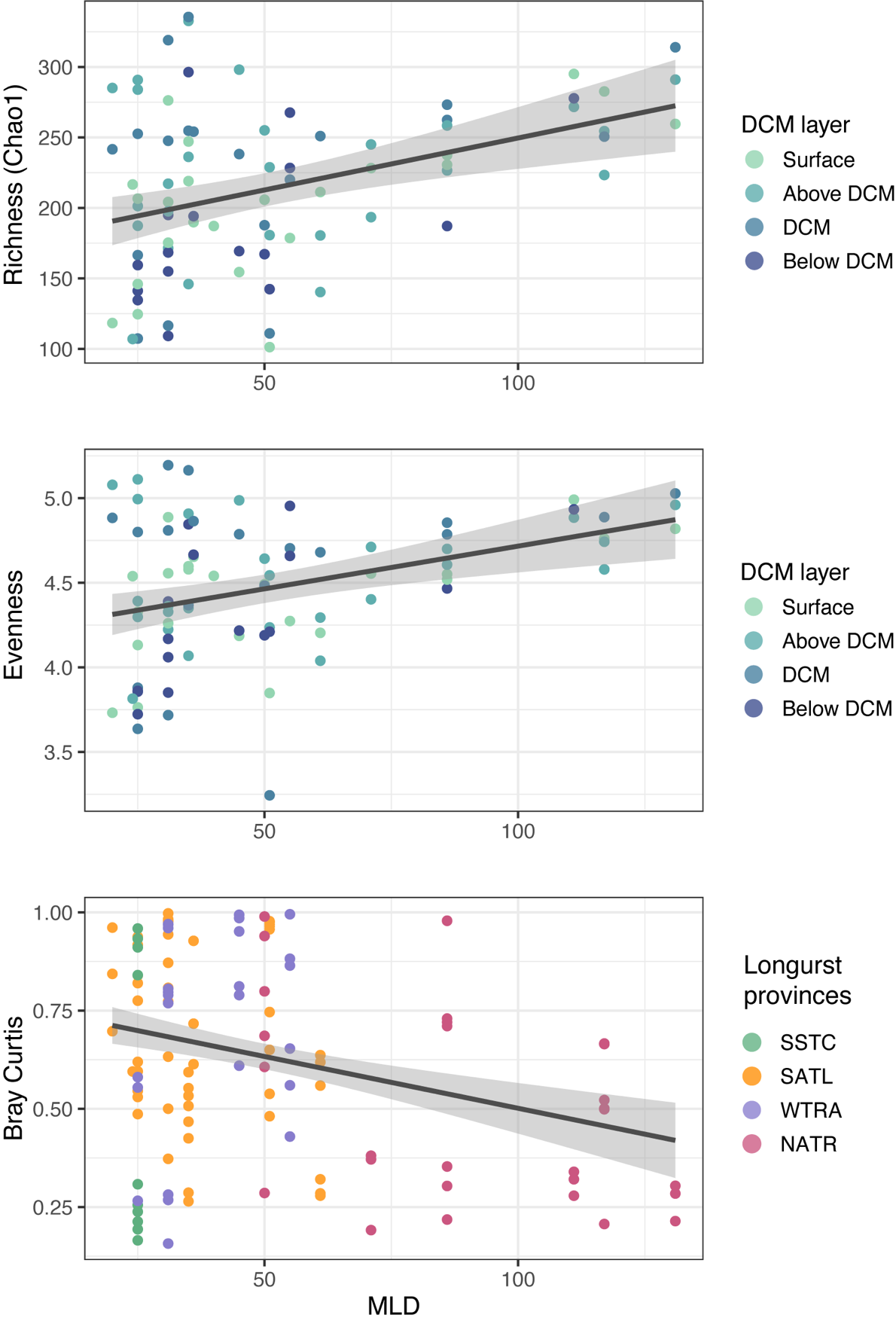


**Figure S4.** Correlation between the mixed layer depth (MLD) and the **A**) Chao1index, **B**) evenness and **C**) beta (Bray-Curtis) diversity index. Only Bray-Curtis values between samples from the same station are displayed. DCM: deep chlorophyll maximum. SSTC, South Subtropical Convergence zone; SATL, South Atlantic gyre; WTRA, Western tropical Atlantic; NATR, North Atlantic Tropical gyre.


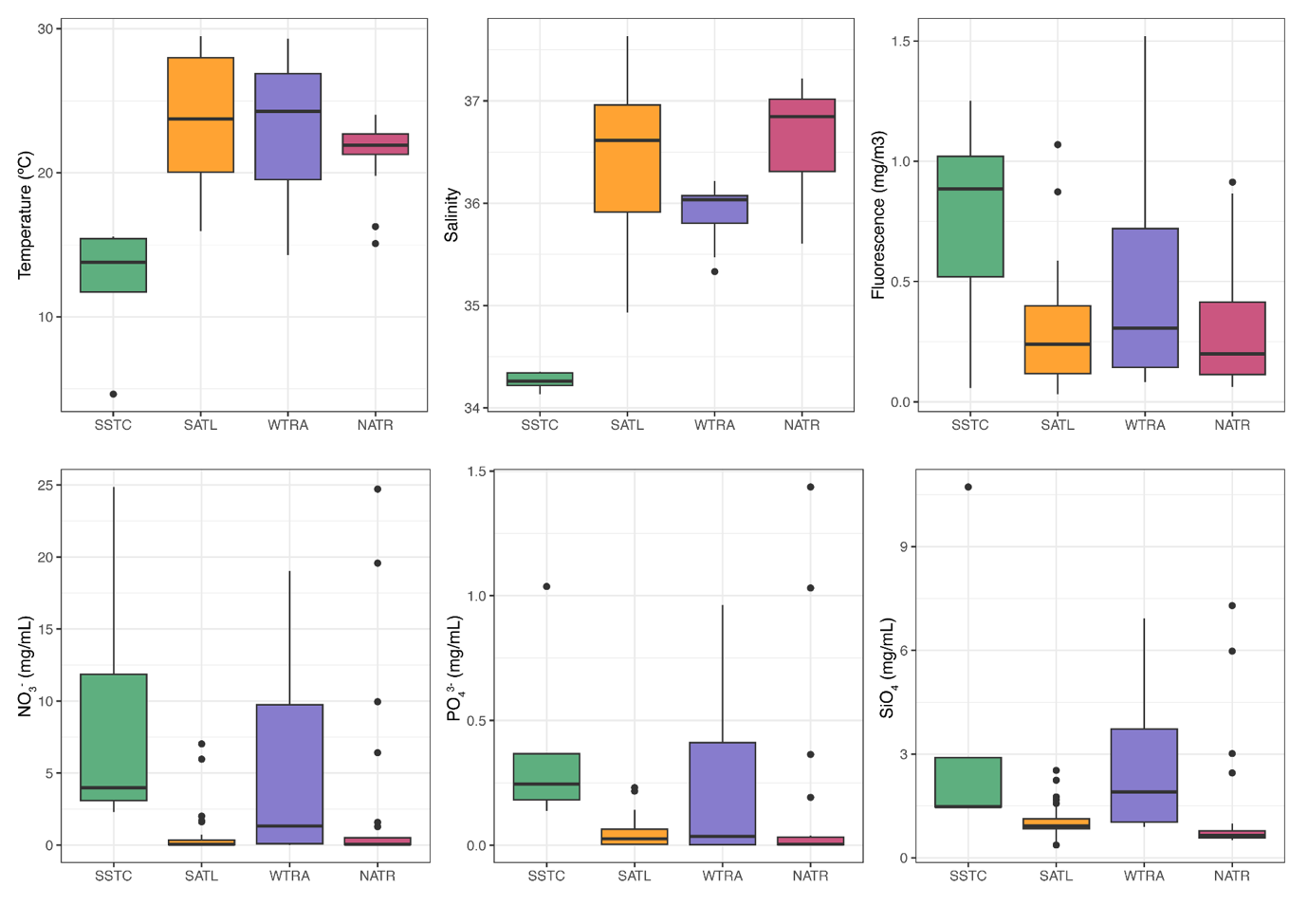


**Figure S5.** Environmental variation at each Longhurst province as seen by the distribution of temperature, salinity, fluorescence, nitrate (NO_3_^-^), phosphate (PO_4_^3-^), and silicate (SiO_4_). SSTC, South Subtropical Convergence zone; SATL, South Atlantic gyre; WTRA, Western tropical Atlantic; NATR, North Atlantic Tropical gyre.

**Figure S6.** Non-metrical multidimensional (nMDS) plots based on the Bray-Curtis dissimilarities between AAP communities, colored by their belonging to the four different Longhurst provinces. SSTC, South Subtropical Convergence zone; SATL, South Atlantic gyre; WTRA, Western tropical Atlantic; NATR, North Atlantic Tropical gyre.


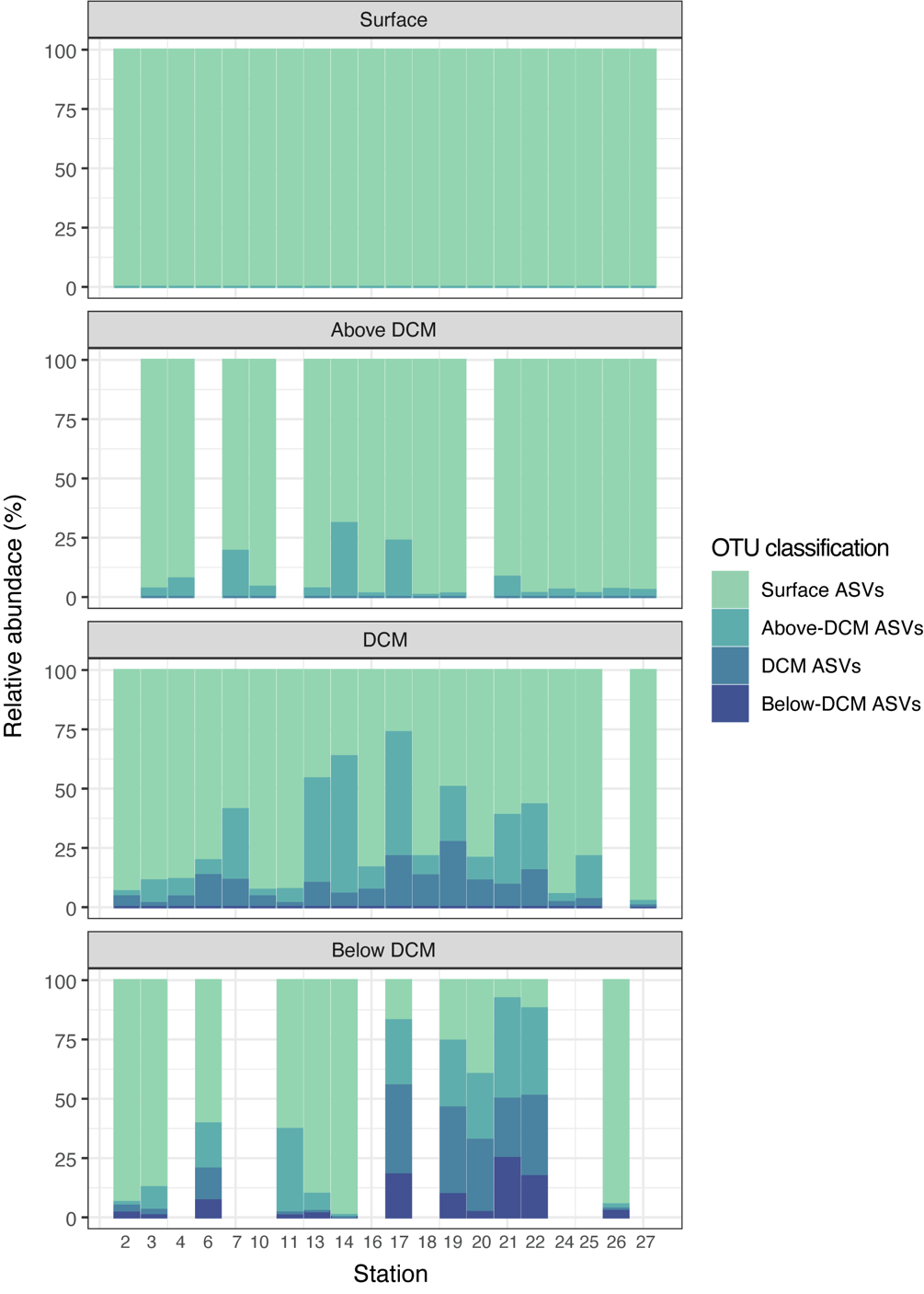


**Figure S7.** Contribution of ASVs categorized as “surface”, “above-DCM”, “DCM” and “below-DCM” in each station and depth. Only stations with three or more depths were included. Each ASV was defined according to the depth where it was first found, starting from the surface and going down.

**
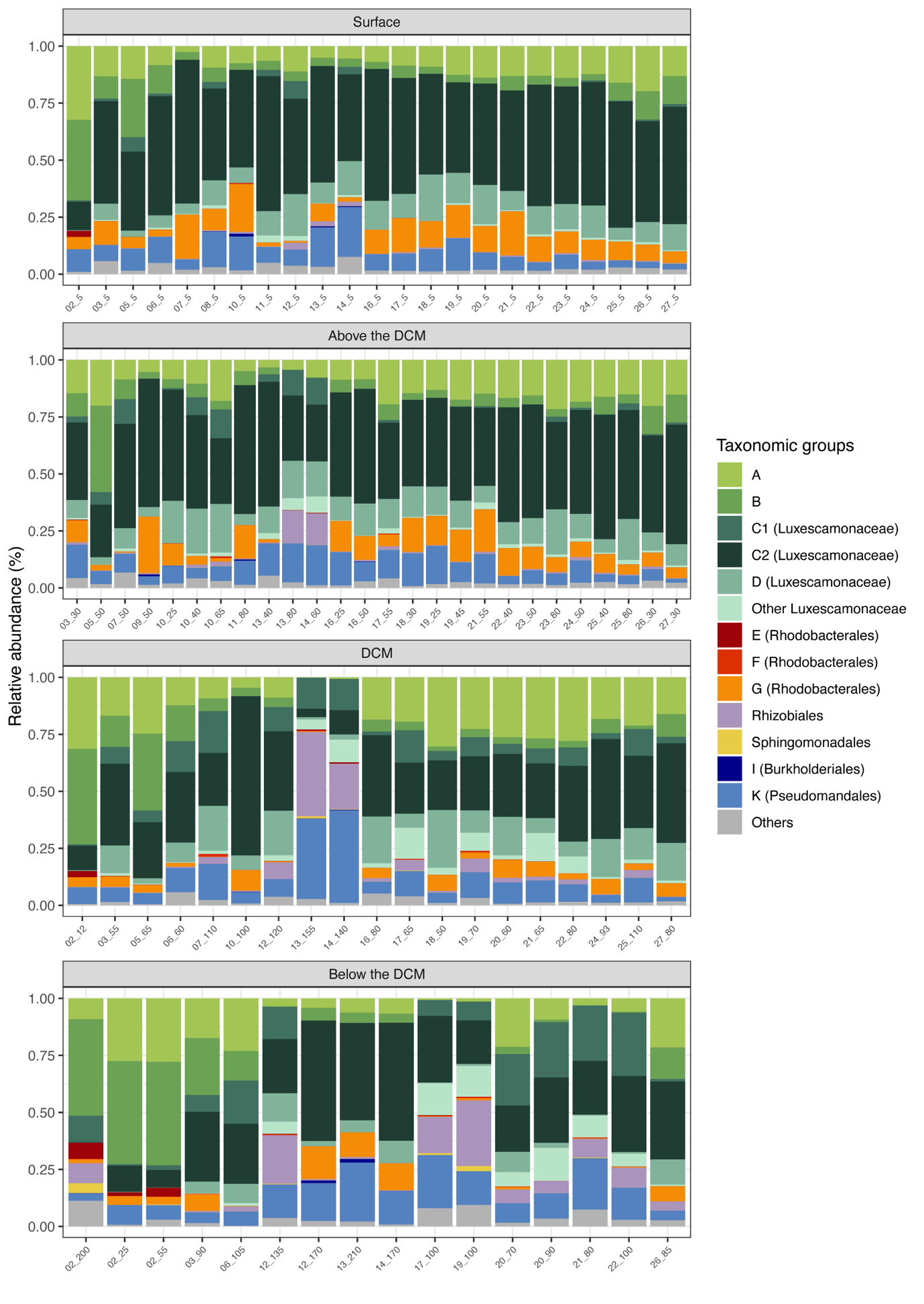
**

**Figure S8.** Taxonomic composition of samples across the Poseidon Expedition, divided by their location across the DCM profile (surface, above the DCM, DCM, or below the DCM). The ID of the samples indicate the station and the depth. E.g. “02_5” indicates that the sample is from station 2 at 5m.

**Table S1**. Result of the *envfit* analysis.

| **Environmental** **variables** | **R2** | **p-value** | **Vector NMDS1** | **Vector NMDS2** |
| --- | --- | --- | --- | --- |
| Depth | 0.19 | 0.002 | - | - |
| Temperature | 0.53 | 0.001 | 0.972618 | -0.23241 |
| Salinity | 0.19 | 0.002 | - | - |
| Oxygen | 0.53 | 0.001 | -0.39092 | -0.92043 |
| Fluorescence | 0.10 | 0.021 | - | - |
| Chlorophyll *a* | 0.05 | 0.134 | - | - |
| NO_2_^-^ | 0.13 | 0.005 | - | - |
| NO_3_^-^ | 0.38 | 0.001 | -0.40664 | 0.913589 |
| SiO_4_ | 0.38 | 0.001 | -0.41573 | 0.909486 |
| PO_4_^3-^ | 0.38 | 0.001 | -0.41851 | 0.90821 |
| *Synechococcus* | 0.16 | 0.001 | - | - |
| *Prochlorococcus* | 0.23 | 0.001 | - | - |
| Latitude | 0.44 | 0.001 | 0.786842 | 0.617154 |

**Table S2**. Mean relative abundance (%) and standard deviation of each taxonomic group.

| **Taxonomic group** | **Mean relative abundance (%)** | **Standard**  **deviation** |
| --- | --- | --- |
| A | 13.51 | 7.80 |
| B | 8.41 | 10.83 |
| C1 (Luxescamonaceae) | 5.04 | 6.63 |
| C2 (Luxescamonaceae) | 37.68 | 14.41 |
| D (Luxescamonaceae) | 9.79 | 6.13 |
| E (Rhodobacterales) | 0.24 | 0.96 |
| F (Rhodobacterales) | 0.10 | 0.21 |
| G (Rhodobacterales) | 6.76 | 5.69 |
| I (Burkholderiales) | 0.09 | 0.28 |
| K (Pseudomonadales) | 10.06 | 6.89 |
| Other Luxescamonaceae | 3.35 | 3.59 |
| Others | 2.71 | 2.05 |
| Rhizobiales | 2.13 | 6.38 |
| Sphingomonadales | 0.13 | 0.54 |
